# Different decision-making in spine metastasis management among radiation oncologists and orthopedic surgeons: a Korean online survey study

**DOI:** 10.3389/fneur.2023.1317858

**Published:** 2024-01-11

**Authors:** Hwa Kyung Byun, Woong Sub Koom, Se-Jun Park, Sang-Il Kim, Jin Ho Kim, Young-Hoon Kim, Bong-Soon Chang, Yong Chan Ahn

**Affiliations:** ^1^Department of Radiation Oncology, Yongin Severance Hospital, Yonsei University College of Medicine, Yongin-si, Republic of Korea; ^2^Department of Radiation Oncology, Yonsei Cancer Center, Yonsei University College of Medicine, Seoul, Republic of Korea; ^3^Department of Orthopedic Surgery, Samsung Medical Center, Sungkyunkwan University School of Medicine, Seoul, Republic of Korea; ^4^Department of Orthopedic Surgery, Seoul St. Mary's Hospital, College of Medicine, The Catholic University of Korea, Seoul, Republic of Korea; ^5^Department of Radiation Oncology, Seoul National University Hospital, Seoul National University College of Medicine, Seoul, Republic of Korea; ^6^Department of Orthopedic Surgery, Seoul National University Hospital, Seoul National University College of Medicine, Seoul, Republic of Korea; ^7^Department of Radiation Oncology, Samsung Medical Center, Sungkyunkwan University School of Medicine, Seoul, Republic of Korea

**Keywords:** spine metastasis, surgical resection, radiotherapy, survey, multidisciplinary discussion, pattern of care

## Abstract

**Purpose:**

To understand the current practice of radiation oncologists (ROs) and orthopedic surgeons (OSs) regarding spine metastasis.

**Methods:**

In 2022, an internet-based survey was conducted for ROs and OSs who treat spinal metastasis in Korea. Respondents were asked to choose the treatment option for two clinical scenarios. Scenario 1 involved a case displaying symptoms of leg weakness due to spinal cord compression and Scenario 2 involved a case with back pain due to pathologic compression fracture. The survey also included a question that required respondents to rank the importance of 11 clinical factors that affect treatment decisions.

**Results:**

Forty-nine ROs and 30 OSs responded to the survey. There were significant differences in treatment choices between two groups for both scenarios (*P* = 0.001). In Scenario 1, more OSs chose surgical resection than ROs (43.3% vs. 16.7%), while more ROs chose radiotherapy than OSs (83.3% vs. 53.3%). In Scenario 2, a similar proportion of OSs and ROs chose radiotherapy (OSs, 71.4% vs. ROs, 67.3%), while more OSs opted for prophylactic fixation after radiotherapy than ROs (95.0% vs. 42.4%). The top three factors influencing treatment decisions were general performance status, life expectancy, and spinal instability for both ROs and OSs. In both Scenarios 1 and 2, the treatment decisions of ROs changed significantly when clinical conditions related to these top three factors were altered.

**Conclusion:**

Although ROs and OSs share the same factors influencing treatment decisions for spinal metastases, notable differences exist in their actual treatment preferences, with ROs preferring radiotherapy and OSs opting for surgical resection. Multidisciplinary discussions may be necessary to reduce the gap in decision-making.

## Introduction

The spine is one of the most common sites of cancer metastasis (1). Spine metastasis can cause symptoms such as pain, spinal cord compression, hypercalcemia, and pathologic fracture, which usually adversely impact the patients' health status and quality of life (2). The clinical situations in which spine metastasis occurs vary greatly from patient to patient, and frequently present with complex therapeutic challenges. The type of cancer, patient's condition, location of involved spine, severity of clinical symptoms metastasis can vary depending on the individual basis. Thus, treatment plans need to be carefully made taking the individual clinical situation of each patient into account.

For managing the spine metastasis patients, various options including analgesics, systemic chemotherapy, hormonal therapy, bisphosphonates, systemic radiopharmaceuticals, and local therapies such as surgery and radiation therapy (RT) are available, and interdisciplinary care among radiologists, medical oncologists, radiation oncologists (ROs), orthopedic surgeons (OSs), pain medicine specialists, and palliative care professionals is desirable (3). Among several options, local therapy is particularly important as it can lead to local tumor control, neural compression relief, and pain control. Surgery has the capability of stabilizing mechanical instability in addition. Thus, it is crucial to understand the current practice patterns by ROs and OSs, who are frequently engaged in the management of spine metastasis patients.

There were two survey studies, initiated by Korean ROs previously, on the practice patterns for spine metastasis, which were limited among ROs (4, 5). Considering that not only ROs but also OSs are main specialties of local therapy for patients with spine metastasis, it would be clinically meaningful to compare and analyze the opinions of two main specialists. We intended, through this study, to investigate the current practice patterns by ROs and OSs in managing the spine metastasis patients and identify the clinical factors influencing their clinical decision-making.

## Materials and methods

### Participants and survey

This study was initiated by the Korean Society of Spine Tumor, and the questionnaires were distributed among the members of three societies: the Korean Society of Spine Tumor; the Korean Society for Radiation Oncology; and the Korean Society of Spine Surgery, respectively. The survey was conducted as an online survey using the Google Forms from August until November 2022. A total of 79 specialists responded to the questionnaire, including 49 ROs and 30 OSs, all of whom were affiliated at the university hospitals or tertiary hospitals.

### Questionnaires

**Figure 1:**
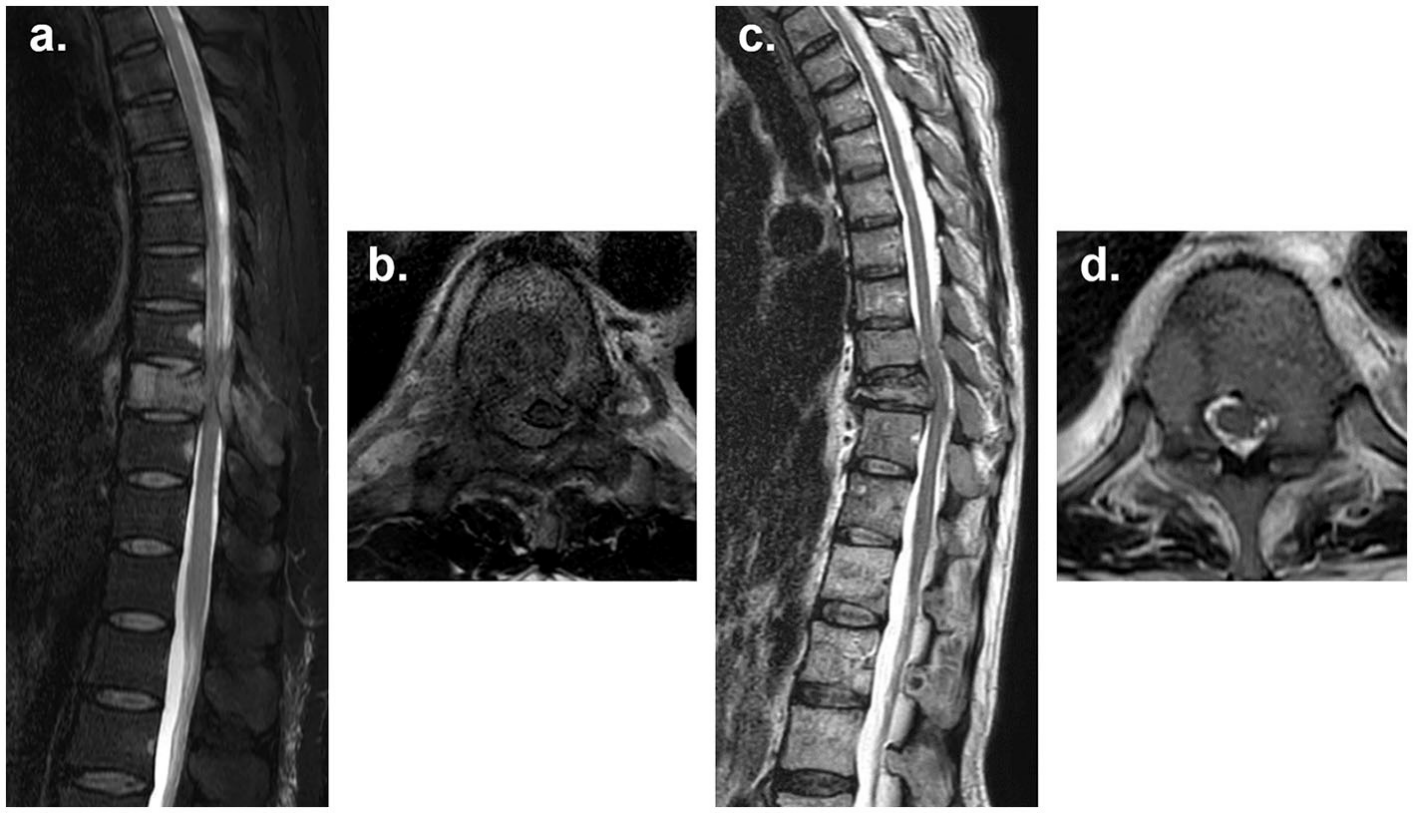
**(a, b)** T2-weighted MR images demonstrate a spine metastasis in clinical scenario 1. Scenario 1 describes a 55-year-old male patient with hepatocellular carcinoma who presented with a week-long history of leg weakness due to spinal cord compression (Bilsky grade 3) at the T7 level. The patient had a single spinal metastasis, a SINS score of 9, a KPS score of 70, an expected survival time of <6 months, and no further plan for systemic treatment. **(c, d)** T2-weighted MR images demonstrate spine metastases in clinical scenario 2. Scenario 2 describes a 60-year-old female patient with breast cancer who presented with severe back pain due to a pathologic compression fracture at the T10 level with spinal cord abutment (Bilsky grade 1c). The patient had multiple spinal metastases, a SINS score of 9, a KPS score of 90, an expected survival time of more than 6 months, and a plan for further systemic treatment.

The questionnaires were developed by consensus among four ROs (HB, WK, JK, and YA) and two OSs (S-JP and S-IK). The survey consisted of three parts. The first was related to the general characteristics of the respondents. In the second part, two clinical scenarios were generated, on each of which, the respondents were asked to choose their preferred treatment option from a list of six: (1) surgical resection alone; (2) surgical resection plus postoperative RT; (3) RT alone; (4) RT plus prophylactic fixation; (5) non-surgical intervention; and (6) observation, respectively. The original clinical scenario 1 described a 55-year-old male patient with hepatocellular carcinoma who presented with a week-long history of leg weakness due to spinal cord compression with Bilsky grade 3 compression at the T7 level (Figures 1a, b) (6). Based on the imaging studies, this patient had a single spine metastasis, and the spinal instability neoplastic score (SINS) was 9, indicating a potentially unstable spine (7). The patient's Karnofsky performance status (KPS) before the onset of leg weakness was 70, with the expected survival time shorter than 6 months. There was no further plan of systemic treatment. The original clinical scenario 2 described a 60-year-old female patient with breast cancer who presented with severe back pain due to pathologic compression fracture at T10 level with spinal cord abutment (Figures 1c, d) (Bilsky grade 1c). The patient's SINS was 9, indicating a potential unstable spine. Multiple spine metastases were also present, and KPS was 90, with the expected survival time longer than 6 months. There was a further plan of systemic treatment. In addition to the original scenarios, the respondents were asked to choose their preferred treatment option assuming that some conditions were different from the original ones while all others were the same (i.e., differences in the involved spine level, life expectancy, SINS, KPS, plan of systemic therapy, etc…). The third part of the survey intended to identify the factors that influenced treatment decision and included 11 factors: patient's age; general performance status; life expectancy; expected complication by spine metastasis-directed treatment; patient's convenience by treatment; expected efficacy of treatment for metastasis; policy and situation of the respondent's institute or training habits; spinal instability; location of the tumor; number of spine metastases; and further systemic treatment plan, respectively. The respondents were asked to rank the factors in the order of importance. The detail of questionnaire is provided in Supplementary material.

### Statistical analysis

The collected answers for the second part by ROs and OSs were compared using the chi-square test. The McNemar's test was conducted to compare whether a significant difference occurred in the modified clinical scenarios, when compared with the original. The Bonferroni correction was applied to correct for multiple comparisons. A two-sided *P*-value <0.05 was considered significant. For the third part, the final ranking was determined by arranging the average value of each respondent's ranking for each clinical factor in order. All the statistical analyses were performed using the SPSS software version 25.0 (IBM Inc., Armonk, NY, USA).

## Results

### Characteristics of respondents

Of the 79 respondents, 30 were OSs and 49 were ROs. The OSs were working in 26 different tertiary or university hospitals, with a median of one respondent per hospital (range, 1–2). The ROs were working in 33 different tertiary or university hospitals, with a median of one respondent per hospital (range, 1–8). Regarding the OSs, the median period of practicing as an orthopedic surgeon was 12 years [interquartile range (IQR), 7–17 years]. The majority (70.0%) reported that >200 spine surgeries were performed per year, and also the majority (66.7%) reported that ≤ 30 surgeries in relation with spine metastasis were performed per year at their departments, respectively (Table 1). Among the ROs, the median period of practicing as a radiation oncologist was 9 years (IQR, 5–17 years). The number of daily RT patients varied from ≤ 50 to >300, and the number of patients who received spine RT per year varied from ≤ 50 to >300 at their departments.

**Table 1:** Characteristics of respondents.

	***N* or median**	**% or IQR**
**Orthopedic surgeons**		
Age (years)	44	40–50
**Sex**		
Female	0	0.0
Male	30	100.0
**No. of annual patients who received spine surgery in**		
**the department**		
51–100	5	16.7
101–200	4	13.3
>200	21	70.0
**No. of annual patients who received spine surgery due to**		
**spine metastasis in the department**		
≤ 30	20	66.7
31–50	7	23.3
51–100	3	10.0
**No. of co-workers as spine surgeon specialists**		
1	7	23.3
2	15	50.0
3–5	8	26.7
Period of practicing orthopedic surgery specialist, years	12	7–17
**Radiation oncologists**		
Age (years)	42	36–48
**Sex**		
Female	14	28.6
Male	35	71.4
**No. of daily patients who received radiotherapy in**		
**the department**		
≤ 50	8	16.3
51–100	17	34.7
101–200	8	16.3
201–300	1	2.0
>300	14	28.6
**No. of annual patients who received radiotherapy to spine in**		
**the department**		
≤ 50	10	20.4
51–100	13	26.5
101–200	12	24.5
201–300	2	4.1
>300	12	24.5
1	5	10.2
2	6	12.2
3–5	20	40.8
6–10	7	14.3
>10	11	22.4
Period of practicing radiation oncology specialist (years)	9	5–17

### Choice of treatment method

**Figure 2:**
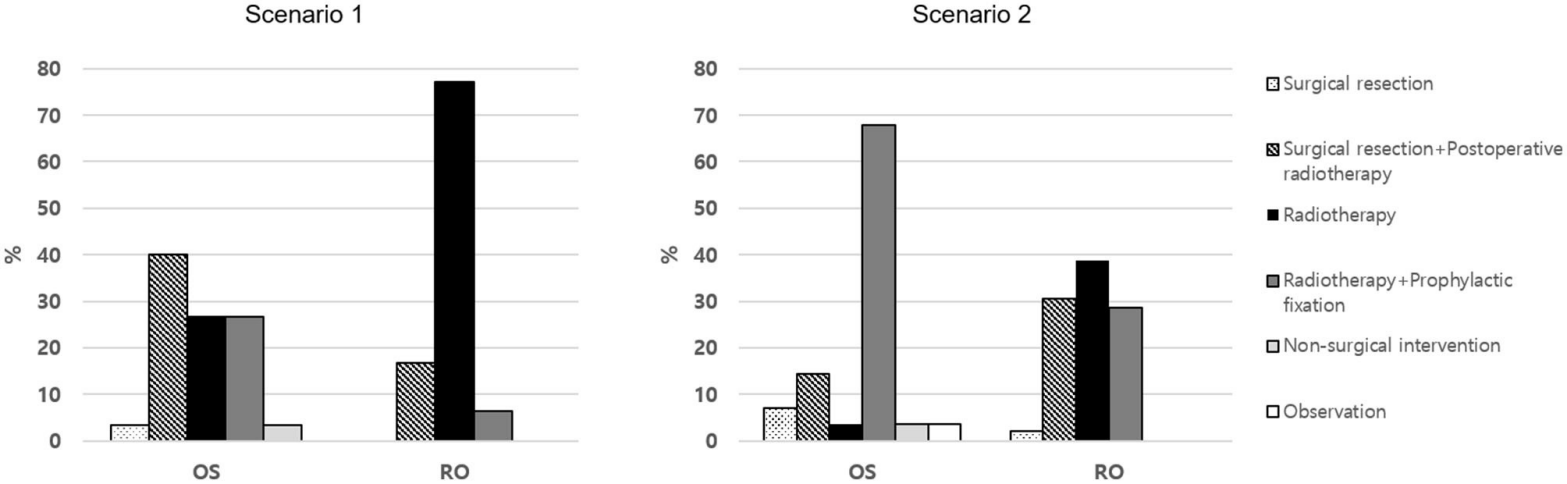
The choice of treatment methods for spine metastasis in clinical scenarios.

In scenario 1, which represented a case with cord compression symptoms, there was a significant difference in treatment choice between OSs and ROs (Figure 2, *P* < 0.001). The rate of choosing surgical resection (with or without postoperative RT) was significantly higher among OSs than ROs (43.3% vs. 16.7%, *P* = 0.010). Conversely, ROs more frequently preferred RT (with or without prophylactic fixation) than OSs (83.3% vs. 53.3%, *P* = 0.004). Among those who chose RT, the proportion of those who chose prophylactic fixation was higher among OSs (50.0% vs. 7.5%, *P* = 0.001).

In scenario 2, which represented a case with pathologic compression fracture, there also was a significant difference in treatment choice between OSs and ROs (Figure 2, *P* < 0.001). RT (with or without prophylactic fixation) was the most frequently chosen treatment method by both OSs and ROs (71.4% vs. 67.3%, *P* = 0.710). Among those who chose RT, however, the proportion of OSs who opted for prophylactic fixation was significantly higher (95.0% vs. 42.4%, *P* < 0.001).

### Changes in treatment methods according to modified clinical conditions

Table 2 summarized the patterns of answers by OSs and ROs on the scenarios 1 and 2. In scenario 1, OSs chose surgical resection in 43.3% of cases in the original scenario, and more frequently chose surgical resection under the condition that the expected survival was longer than 6 months (76.7%, *P* = 0.018), but their choices did not change on other modified conditions. Among ROs, 16.7% chose surgical resection in the original scenario, and more frequently chose surgical resection if the expected survival was longer than 6 months (51.0%, *P* < 0.001), if there was an unstable spine (49.0%, *P* < 0.001), if the KPS was 90 (44.9%, *P* = 0.009), and if symptom duration was 1 day (51.0%, *P* < 0.001), respectively.

**Table 2:** Number of answers selecting surgical resection according to the clinical conditions.

	**Orthopedic surgeons' answer**			**Radiation oncologists' answer**		
	**Surgical resection** ***N*** **(%)**	**Other** ***N*** **(%)**	***P*** **-value** ^*^	**Surgical resection** ***N*** **(%)**	**Other** ***N*** **(%)**	***P*** **-value** ^*^
Scenario 1—Cord compression	13 (43.3)	17 (56.7)	–	8 (16.7%)	40 (83.3)	–
C spine location	14 (50.0)	14 (50.0)	>0.999	10 (20.4)	39 (79.6)	>0.999
Expected survival >6 months	23 (76.7)	7 (23.3)	0.018	25 (51.0)	24 (49.0)	<0.001
Stable spine	12 (40.0)	18 (60.0)	>0.999	8 (16.3)	41 (83.7)	>0.999
Unstable spine	16 (53.3)	14 (46.7)	>0.999	24 (49.0)	25 (51.0)	<0.001
KPS 90	17 (58.6)	12 (41.4)	0.567	22 (44.9)	27 (55.1)	0.009
KPS 50	4 (13.8)	25 (86.2)	0.072	1 (2.1)	47 (97.9)	0.144
Symptom duration 1 day	16 (53.3)	14 (46.7)	>0.999	25 (51.0)	24 (49.0)	<0.001
Symptom duration 1 month	9 (30.0)	21 (70.0)	>0.999	7 (14.3)	42 (85.7)	>0.999
Further systemic treatment plan	16 (53.3)	14 (46.7)	>0.999	17 (34.7)	32 (65.3)	0.108
Scenario 2—Pathologic compression fractures	6 (21.4)	22 (78.6)	–	16 (32.7)	33 (67.3)	–
C spine location	10 (35.7)	18 (64.3)	>0.999	19 (38.8)	30 (61.2)	>0.999
L spine location	6 (21.4)	22 (78.5)	>0.999	16 (32.7)	33 (67.3)	>0.999
Expected survival <6 months	3 (10.7)	25 (89.3)	>0.999	1 (2.0)	48 (98.0)	<0.001
Stable spine	0 (0.0)	29 (100.0)	NA	8 (16.3)	41 (83.7)	0.312
Unstable spine	7 (24.1)	22 (75.9)	>0.999	27 (55.1)	22 (44.9)	0.024
KPS 70	4 (13.8)	25 (86.2)	>0.999	11 (22.4)	38 (77.6)	0.504
KPS 50	4 (13.8)	25 (86.2)	>0.999	3 (6.1)	46 (93.9)	0.002
Mild pain (VAS 3–4)	0 (0.0)	29 (100.0)	NA	8 (16.3)	41 (83.7)	0.168

^*^P values were obtained by comparing the original scenario with the case where each clinical condition was changed from the original scenario. Bonferroni-adjusted P values are presented.

KPS, the Karnofsky Performance Status score.

In scenario 2, 21.4% of OSs chose surgical resection in the original scenario, and no one chose surgical resection if the spine was stable or if the patient had mild pain. Among ROs, 32.7% chose surgical resection in the original scenario, which was significantly less frequently chosen if the expected survival was shorter 6 months (2.0%, *P* < 0.001) or if the KPS score was 50 (6.1%, *P* = 0.002), respectively. On the other hand, ROs chose surgical resection more frequently if the spine was unstable (55.1%, *P* = 0.024).

### The ranking of factors influencing treatment decisions

In the third part, the factors influencing treatment decisions were ranked by the participating OSs and ROs (Table 3). The top three factors chosen by ROs were general performance status, followed by life expectancy, and spinal instability, respectively. Those chosen by OSs were life expectancy, followed by general performance status, and spinal instability, respectively. The top three factors were, more or less, very similar between specialties. Both groups also commonly considered the expectation of treatment efficacy for spine metastasis and the number of spine metastasis as an important factor, with five out of the top six factors being in common.

**Table 3:** Ranking of clinical factors influencing treatment decision-making for spine metastasis.

**Radiation oncologists' answer**		**Orthopedic surgeons' answer**	
**Rank**	**Factors**	**Rank**	**Factors**
1	General performance status	1	Life expectancy
2	Life expectancy	2	General performance status
3	Spinal instability	3	Spinal instability
4	Number of spine metastasis	4	Expectation of efficacy of treatment for spine metastasis
5	Expectation of efficacy of treatment for spine metastasis	5	Patient age
6	Location of the tumor	6	Location of the tumor
7	Patient age	7	Further systemic treatment plan
8	Further systemic treatment plan	8	Complications of treatment for spine metastasis
9	Complications of treatment for spine metastasis	9	Patient convenience in the treatment
10	Patient convenience in the treatment	10	Number of spine metastasis
11	Policy and situation of your department or training habits	11	Policy and situation of your department or training habits

## Discussion

In this study, we aimed to investigate the current practice patterns of the Korean ROs and OSs with respect to the preferred management option for the spine metastasis patients and the factors influencing their treatment decisions. Our findings demonstrated significant differences between the two specialties, with ROs predominantly favoring RT while OSs preferring surgical resection, respectively. Despite this divergence in treatment preferences, the top three factors influencing the treatment decision (general performance status, life expectancy, and spinal instability) were in common among the two specialties. Notably, in both Scenarios 1 and 2, the treatment decisions of ROs showed significant differences when clinical conditions related to these top three factors were altered.

The results of this study highlight that there exists significant heterogeneity in the practice patterns across two different clinical backgrounds: OSs tend to favor surgical intervention; while ROs generally prefer RT, respectively. This could be attributed to the heterogeneous nature of bone metastasis and the availability of diverse treatment options. Numerous methods have been proposed to aid treatment decision-making for spine metastasis. The SINS aims to quantify the spinal instability and to assist in surgical decision-making (7). The modified Tokuhashi scoring system strives to predict prognosis in the patients with metastatic spinal disease using six factors: general condition; number of non-spinal bone metastasis; number of spinal metastasis; type of primary lesion; presence or absence of metastasis to major organs; and state of paralysis, respectively (8). The Tomita's scoring system is based on the type of primary tumor, presence and operability of visceral metastasis, and number of bony metastasis, respectively (9). In the guidelines for the diagnosis and treatment of bone metastasis published by Japanese medical groups, surgery and RT are suggested as local therapy options (10). However, this guideline offered limited details regarding the selection of specific local therapies in various clinical situations. No universal guideline, which considers all patient-, tumor-, and spine-related clinical elements, is available as of yet. In this context, examining the experts' clinical decision-making in the representative clinical scenarios through surveys, as in this study, could be beneficial and valid to understanding the current real-world practice patterns.

A randomized trial conducted by Patchell et al. (11) demonstrated that direct decompressive surgical resection followed by RT provided better outcomes in terms of preserving or improving ambulatory function, local control, and survival, when compared to RT alone. However, the application of surgery is not always available because of inherent risks of complications (12). Hence, it is crucial to make an appropriate selection of the patients who require surgery based on their clinical condition. In our study, we examined whether the changes in clinical conditions within the clinical scenarios influenced surgical decision-making. As a result, surgical treatment was preferred when the expected survival was longer, general performance status was better, spine was unstable, and symptom onset of spinal cord compression was recent, respectively. On the other hand, non-surgical treatment was more frequently favored when the expected survival was shorter, general performance status was poorer, spine was stable, and there was only mild pain, respectively. Additionally, the top three important clinical factors in treatment decisions were in common between ROs and OSs (life expectancy, general performance status, and spinal instability).

Our study could add to the existing literature, which were primarily focused on surveys of single specialties. Yu et al. (5) previously surveyed the practice patterns of Korean ROs in the context of spine metastasis. A multinational online survey study, encompassing three Asian countries (Korea, China, and Japan), showed diverse practice preferences in RT for spine metastasis in each nation (4). Spratt et al. (13) examined a real world practice pattern of the U.S. ROs in treating bone metastasis, showing diverse RT approaches. In contrast to the previous studies that solely investigated RT, our study assessed both RT and surgery as local therapies for spine metastasis. This multidisciplinary approach would offer a more comprehensive understanding of clinical practice patterns in this field.

The limited number of participating physicians in this survey represents a significant limitation of this study. However, this survey was sent to ROs and OSs nationwide, and most of the participants belonged to the tertiary or university hospitals in Korea and their main interest practice fields include spine tumor. Another limitation is the presentation of a limited number of scenarios. Nevertheless, within each scenario, variations in clinical conditions were introduced to facilitate the consideration of treatment decisions in a broader range of situations.

The discrepancy in treatment decision shown between ROs and OSs in our study calls for the need for multidisciplinary care. Harel and Angelov (14) suggested a treatment algorithm for the patients with spine metastasis incorporating RT, radiosurgery, systemic therapy, and surgery, emphasizing multidisciplinary integration. Barton et al. (15) interviewed a multidisciplinary cohort of physicians treating spine metastasis, in which, the respondents strongly emphasized the importance of multidisciplinary care and communication among multidisciplinary team members. To achieve a multidisciplinary approach, Kimura (16) suggested a cancer board focusing on the management of bone metastasis involving a team of doctors in oncology, palliative care, radiation oncology, orthopedics, nuclear medicine, radiology, and rehabilitation, respectively. In Korea, however, it is known that only a few hospitals operate regular multidisciplinary tumor boards specialized in bone metastasis. The shared-decision making through the specialized cancer board will contribute to improving the care quality, patient satisfaction level as well as the clinical outcomes. More active participation among the specialists who care for the spine tumor patients are highly encouraged.

## Conclusions

Our study suggests that the actual decision-making processes differ significantly between ROs and OSs, although both parties take into account the factors in common in their treatment decision (general performance status, life expectancy, spinal instability, expectation of treatment, and number of spine metastasis). This implies that the same patient is more likely to receive RT if seen by ROs first, and more likely to undergo surgery if seen by Oss first, respectively. This discrepancy in treatment policy may cause confusion to the patients and may interfere with obtaining the optimal treatment outcomes. Our findings indicate that it is necessary to engage in multidisciplinary discussion and determine treatment plan based on the individual clinical situation in order to reduce the gap in treatment decision and to endorse higher clinical benefit to the patients.

## Data availability statement

The raw data supporting the conclusions of this article will be made available by the authors, without undue reservation.

## Author contributions

HB: Data curation, Formal analysis, Methodology, Writing—original draft. WK: Conceptualization, Data curation, Methodology, Writing—review & editing. S-JP: Data curation, Methodology, Writing—review & editing. S-IK: Data curation, Methodology, Writing—review & editing. JK: Data curation, Methodology, Writing—review & editing. Y-HK: Data curation, Writing—review & editing. B-SC: Data curation, Writing—review & editing. YA: Conceptualization, Data curation, Methodology, Writing—review & editing.
