# Supplementary material for: Different decision-making in spine metastasis management among radiation oncologists and orthopedic surgeons: a Korean online survey study
Source: Front Neurol. 2024 Jan 11;14:1317858. doi: 10.3389/fneur.2023.1317858 (PMC10808525; doi:10.3389/fneur.2023.1317858)
Supplement: Supplementary file 1 [file Data_Sheet_1.pdf]

Supplementary

Questionnaire study for the management of spine metastasis

Part 1. General characteristics

Q1. What is your age? ______________years

Q2. Please select your gender.

1) Female 2) Male

Q3. Please select your department.

1) Orthopedics 2) Radiation Oncology

If you are a specialist in orthopedics, please answer the following questions (Q4~7):

Q4. How many patients received spinal surgery in your department in the last 12 months?

1) ≤ 30 2) 31~50 3) 51~100 4) 101~200 5) >200

Q5. How many patients received surgery for spinal stenosis in your department in the last 12 months?

1) ≤ 30 2) 31~50 3) 51~100 4) 101~200 5) >200

Q6. How many specialists in orthopedic surgery perform spinal surgery in your department?

1) 1 2) 2 3) 3-5 4) 6-10 5) >10

Q7. For how many years have you worked as a specialist in orthopedics? ______________years

If you are a specialist in radiation oncology, please answer the following questions (Q8~11):

Q8. How many patients receive radiation therapy on average per day in your department?

1) ≤ 50 2) 51~100 3) 101~200 4) 201~300 5) >300

Q9. How many patients received radiation therapy for spinal cord in your department in the last 12 months?

1) ≤ 50 2) 51~100 3) 101~200 4) 201~300 5) >300

Q10. How many specialists in radiation oncology work in your department?

1) 1 2) 2 3) 3-5 4) 6-10 5) >10

Q11. For how many years have you worked as a specialist in radiation oncology? ______________years

Part 2. Clinical scenarios

Please select a treatment method to be used in the following case from the options below.

1) Surgical resection alone

2) Surgical resection + postoperative radiotherapy

3) Radiotherapy alone

4) Radiotherapy + prophylactic fixation

5) Non-surgical intervention

6) Observation

Case 1

A 55-year-old male patient with recurrent hepatocellular carcinoma presented with a week-long history of leg weakness. The muscle strength in both legs was grade 3, indicating contraction against gravity only. Spinal MRI revealed a lesion at the T7 level causing spinal cord compression. The degree of spinal cord compression was bilsky grade 3, indicating a spinal cord compression without cerebrospinal fluid visible around the cord). No other metastatic lesions were detected. The patient's Karnofsky Performance status before the onset of leg weakness was 70. The expected survival time was less than 6 months. The SINS score was 9 points, indicating a potential unstable spine. The patient was receiving only supportive care, and there were no plans for additional anticancer treatment in the future.

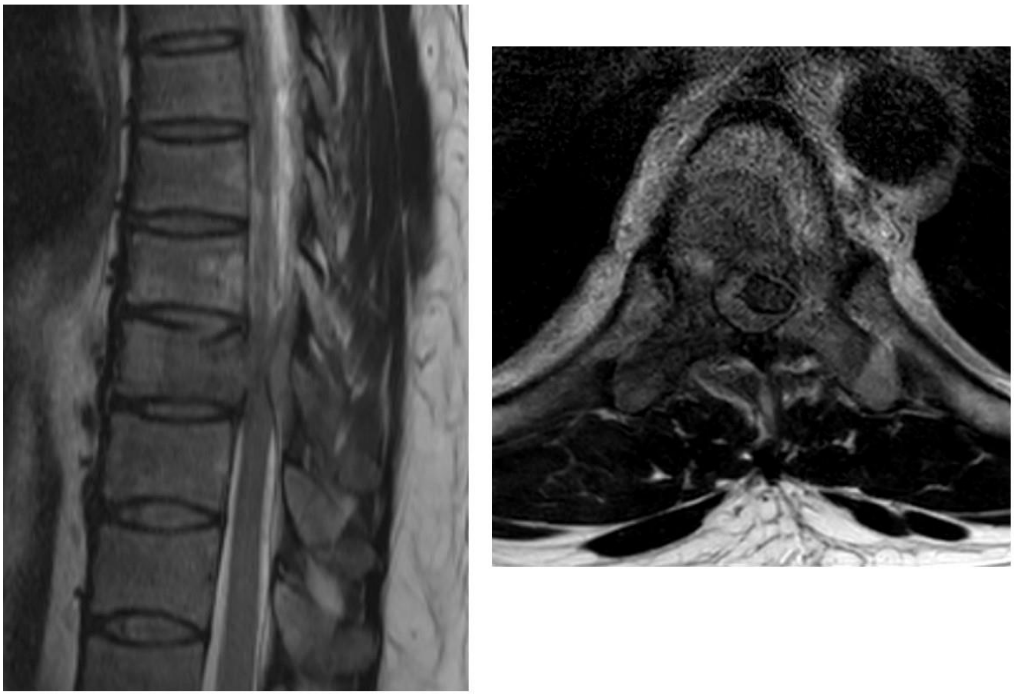

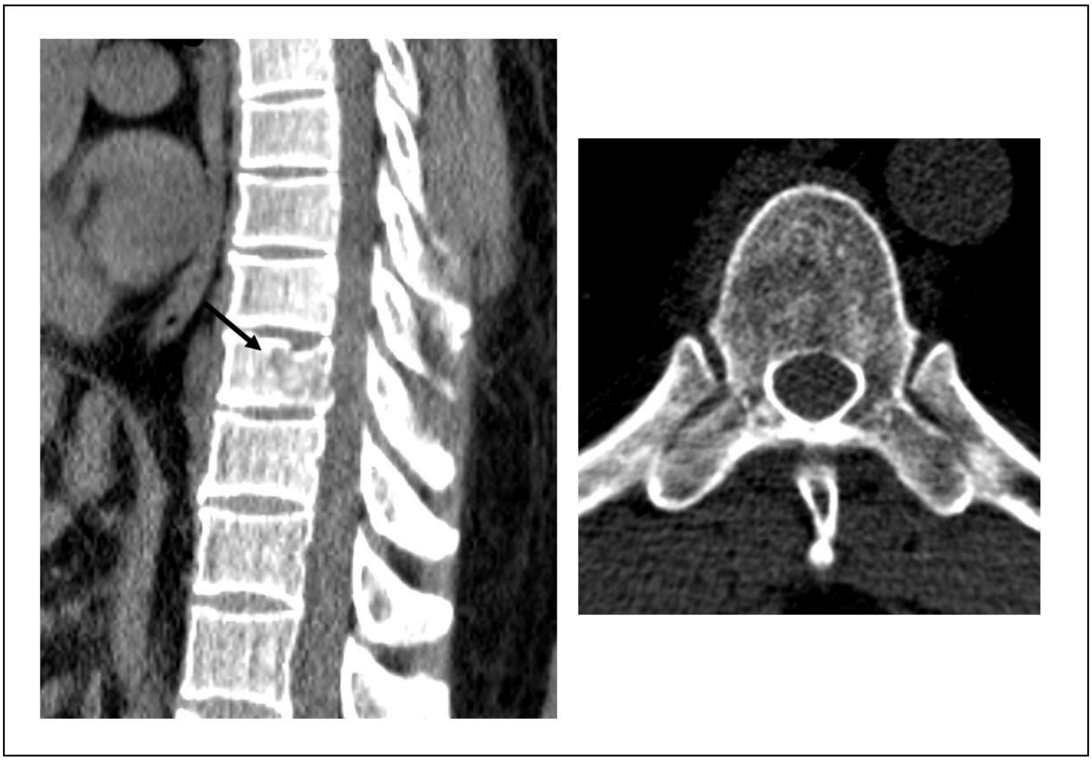

Q1-1. Which treatment method would you choose in the above case?

Q1-2. If the tumor is located at C3-4 in the above case and everything else is the same, which treatment method would you choose?

Q1-3. If the life expectancy is over 6 months in the above case and everything else is the same, which treatment method would you choose?

Q1-4. If the SINS score is 3 points (indicating a stable spine) in the above case and everything else is the same, which treatment method would you choose?

Q1-5. If the SINS score is 14 points (indicating an unstable spine) in the above case and everything else is the same, which treatment method would you choose?

Q1-6. If the symptoms occurred one month ago in the above case and everything else is the same, which treatment method would you choose?

Q1-7. If the symptoms occurred one day ago in the above case and everything else is the same, which treatment method would you choose?

Q1-8. If the Karnofsky Performance status is 90 in the above case and everything else is the same, which treatment method would you choose?

Q1-9. If the Karnofsky Performance status is 50 in the above case and everything else is the same, which treatment method would you choose?

Q1-10. If the patient is receiving or planning to receive anti-cancer treatment and everything else is the same in the above case, which treatment method would you choose?

Case 2

A 60-year-old woman with metastatic breast cancer presented with severe lower back pain (VAS 8-9) that began three days ago. Analgesics did not provide relief of symtom. The patient did not exhibit any other neurological symptoms. Spinal MRI revealed a compression fracture at T10 level with spinal cord abutment (Bilsky grade 1c). Multiple bone metastases were also found. The patient's Karnofsky Performance Status was 90. The expected survival time was more than 6 months. The SINS score was 9, indicating a potential unstable spine. The patient was receiving systemic treatment at the time.

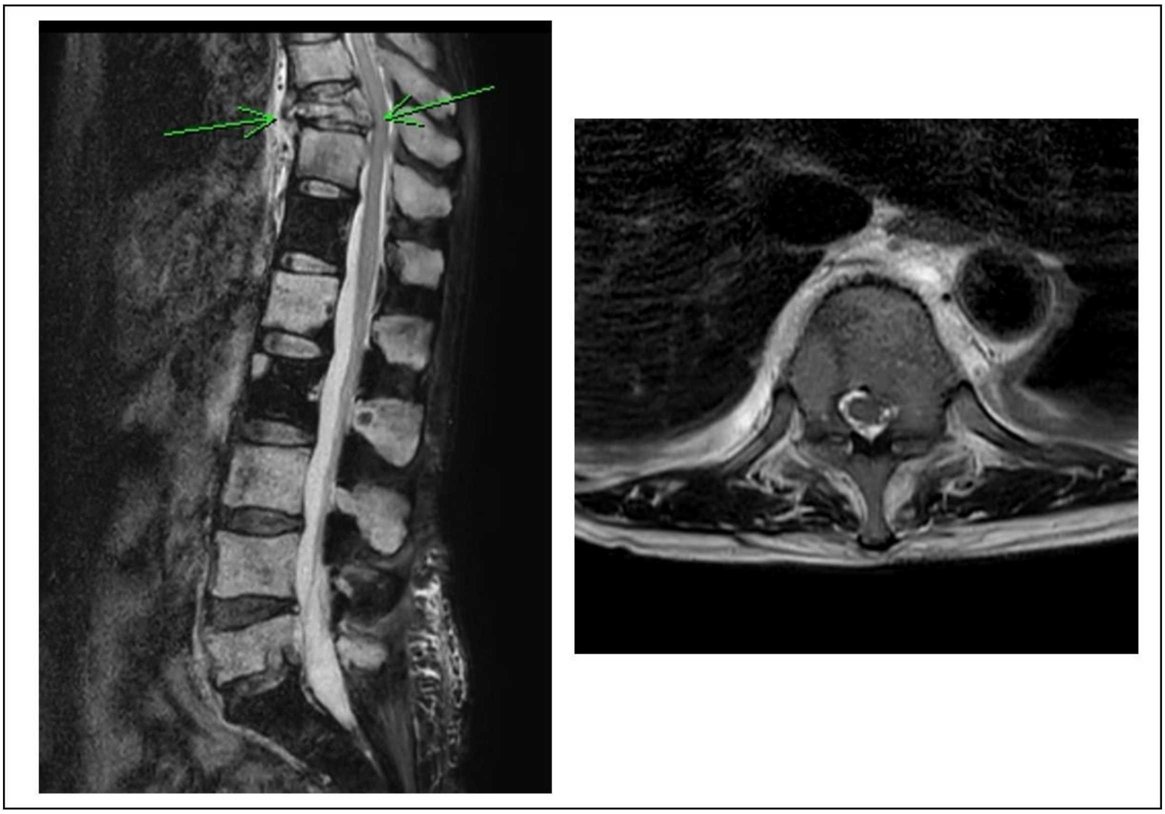

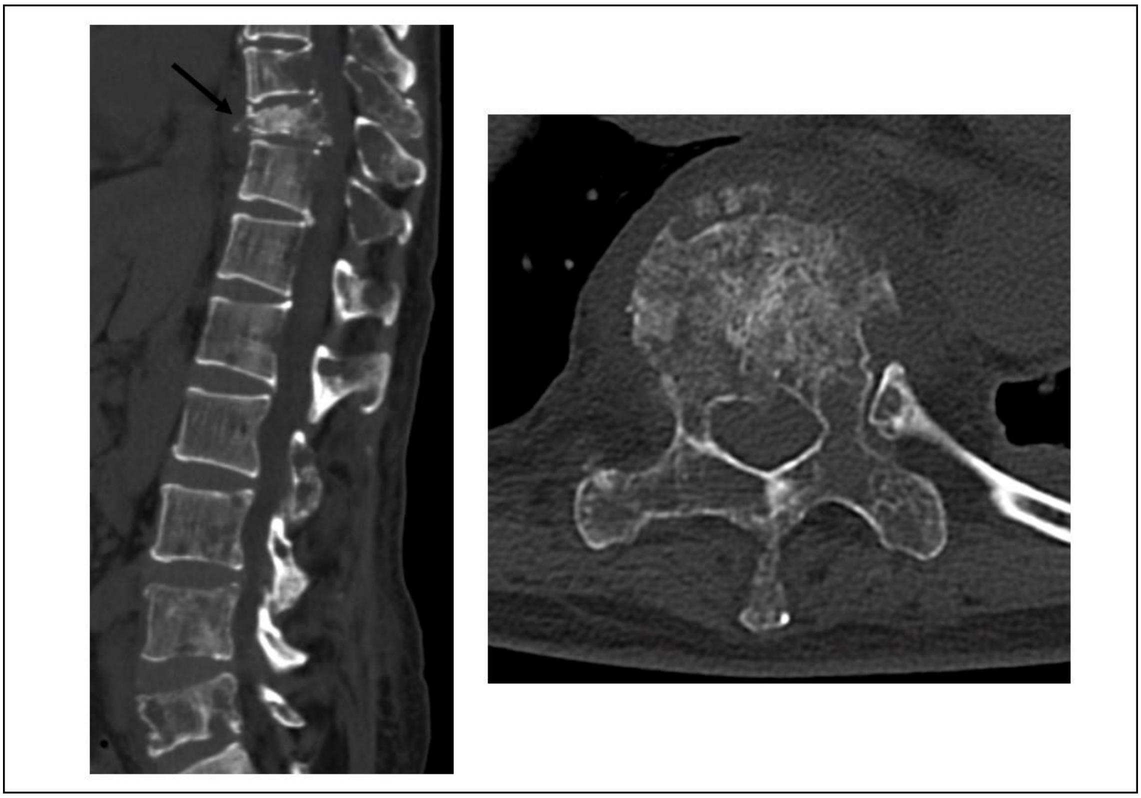

Q2-1. Which treatment method would you choose in the above case?

Q2-2. If the location of the tumor is at C3-4 in the above case and everything else is the same, which treatment method would you choose?

Q2-3. If the location of the tumor is at L3-4 in the above case and everything else is the same, which treatment method would you choose?

Q2-4. If the life expectancy is less than 6 months in the above case and everything else is the same, which treatment method would you choose?

Q2-5. If the SINS score is 3 points (stable spine) in the above case and everything else is the same, which treatment method would you choose?

Q2-6. If the SINS score is 14 points (unstable spine) in the above case and everything else is the same, which treatment method would you choose?

Q2-7. If the Karnofsky Performance status is 70 in the above case and everything else is the same, which treatment method would you choose?

Q2-8. In the above case, if the Karnofsky Performance status is 50 and everything else is the same, what treatment method would you choose?

Q2-9. In the above case, if the degree of pain is mild (VAS 3-4) and everything else is the same, what treatment method would you choose?

Part 3.

The following are 11 clinical factors that may influence the treatment decision for spine metastasis:

1. Patient age
2. General performance status
3. Life expectancy
4. Complications of treatment for spine metastasis
5. Patient convenience in the treatment
6. Expectation of efficacy of treatment for spine metastasis
7. Policy and situation of your department or training habi ts
8. Spinal instability
9. Location of the tumor
10. Number of spine metastases
11. Further systemic treatment plan

Q1. Please list in order from most important to least important the factors you consider when making a treatment decision for spine metastasis. (Example: 9 > 8 > 7 > 10 > 11 > 6 > 5 > 1 > 2 > 3 > 4)
